# Application of machine learning in dementia diagnosis: A systematic literature review

**DOI:** 10.1016/j.heliyon.2023.e21626

**Published:** 2023-11-04

**Authors:** Gauhar Kantayeva, José Lima, Ana I. Pereira

**Affiliations:** Research Centre in Digitalization and Intelligent Robotics (CeDRI), Instituto Politecnico de Bragança, Bragança, Portugal

**Keywords:** 0000, 1111, Machine learning, Dementia, Alzheimer's disease, Neurodegenerative diseases

## Abstract

According to the World Health Organization forecast, over 55 million people worldwide have dementia, and about 10 million new cases are detected yearly. Early diagnosis is essential for patients to plan for the future and deal with the disease. Machine Learning algorithms allow us to solve the problems associated with early disease detection. This work attempts to identify the current relevance of the application of machine learning in dementia prediction in the scientific world and suggests open fields for future research. The literature review was conducted by combining bibliometric and content analysis of articles originating in a period of 20 years in the Scopus database. Twenty-seven thousand five hundred twenty papers were identified firstly, of which a limited number focused on machine learning in dementia diagnosis. After the exclusion process, 202 were selected, and 25 were chosen for analysis. The recent increasing interest in the past five years in the theme of machine learning in dementia shows that it is a relevant field for research with still open questions. The methods used to identify dementia or what features are used to identify or predict this disease are explored in this study. The literature review revealed that most studies used magnetic resonance imaging (MRI) and its types as the main feature, accompanied by demographic data such as age, gender, and the mini-mental state examination score (MMSE). Data are usually acquired from the Alzheimer's Disease Neuroimaging Initiative (ADNI). Classification of Alzheimer's disease is more prevalent than prediction of Mild Cognitive Impairment (MCI) or their combination. The authors preferred machine learning algorithms such as SVM, Ensemble methods, and CNN because of their excellent performance and results in previous studies. However, most use not one machine-learning technique but a combination of techniques. Despite achieving good results in the studies considered, there are new concepts for future investigation declared by the authors and suggestions for improvements by employing promising methods with potentially significant results.

## 1 Introduction

Dementia is a decline in cognition that obstructs daily, domestic, or social functioning in the last decades of life [1]. Studies have shown that risk factors associated with numerous diseases and injuries in the past cause dementia. In contrast, it is attainable to cut back the risk factors through an active lifestyle, avoiding harmful habits, such as smoking and alcohol consumption, healthy nutrition, and weight and blood sugar control.

Being diagnosed early and receiving an accurate rate of progression helps people improve their quality of life and plan their future while making decisions; they can also receive appropriate treatment and care [2].

The diagnosis of dementia cannot be made based on clinical tests alone. Dementia is a diagnosis based on behavior. Diagnosing Alzheimer's disease (AD) or disease variations can only be verified during specific tests and observations [3]. The decision-making process takes an attentive contemplation of medical history, characteristic changes in the thinking process, daily actions, behavior, and psychiatric and laboratory tests. However, recognizing a particular type of dementia is challenging because of the possibility of coinciding with natural aging decline symptoms [4]. An early stage with minor cognitive decline, which may lead to AD or other neurodegenerative diseases, is called Mild Cognitive Impairment (MCI) [5].

According to Alzheimer Yearbook 2019, [6] Europe had 8,885,101 people with dementia in 2018, expected to double by 2050 to 16,276,070. As stated by the Yearbook, the total number of individuals with dementia in the European population is 1.78% (8,885,101 people): the highest rate was indicated in Italy at 2.12%, followed by Greece with 1.99%, Germany at 1.91%, and Portugal with 1.88%, the lowest numbers were detected in Cyprus-1.17%, Slovakia -1.15% and Ireland with 1.09%. The prediction shows that by 2025, the total rate will increase to 2.0% (10,283,905 people): Italy has a prevalence of 2.44% in dementia, Greece 2.27%, and Portugal 2.29% [6].

Providing qualitative data for the determination of dementia and exclusion of other disease symptoms include tests such as Mini-Mental State Examination (MMSE) to measure cognitive impairment; a complete psychiatric test; neuropsychological testing; Computerized Tomography (CT) to exclude brain tumor or subnormal hematoma; Positron Emission Tomography (PET) to measure rate of glucose utilization, oxygen consumption and Magnetic Resonance Imaging (MRI) to create detailed image and Electroencephalography (EEG) [3].

**Figure 1:**
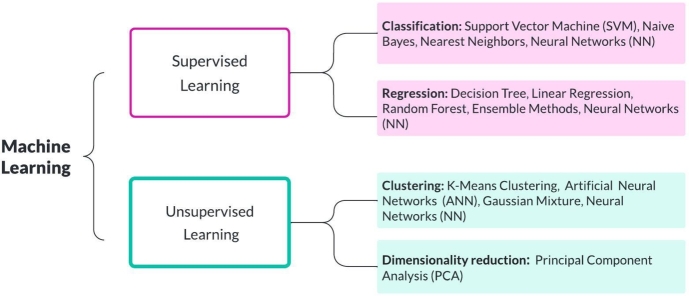
The widely used machine learning algorithms.

Machine Learning (ML), a branch of artificial intelligence (AI), allows us to solve problems such as diagnosis, detection and prediction. ML techniques are based on learning from experience, improving its accuracy by imitating human thinking processes [7]. The major ML tasks are supervised and unsupervised learning. Supervised algorithms need assistance and learn the patterns from training datasets that allow them to be applied to test datasets to classify or predict the output. The widely used supervisor algorithms are the Support Vector Machine (SVM), Decision Tree and Naive Bayes, Linear Regression, Random Forest (RF), and Neural Networks (NN). Unlike previous algorithms, unsupervised learning does not require human assistance and provides no correct answers. This method can be used to discover new patterns independently. The main techniques are K-Means Clustering, Artificial Neural Networks (ANN), Gaussian Mixture, Neural Networks (NN), and Principal Component Analysis (PCA). A quick look at the most commonly used ML algorithms is shown in Fig. 1 [8].

The application of ML for diagnosis detection yielded decent results with high accuracy, as presented in the studies below. Implementing various machine learning algorithms, such as Support Vector Machine (SVM), Convolutional Neural Networks (CNN), Random Forest (RF), and algorithm conjunctions provided a high accuracy, approximately 90% and greater, primarily for the classification of Alzheimer's disease from patients with Mild Cognitive Impairment (MCI) or Cognitive Normal (CN) patients, then identify and discover new disease-related patterns.

This study attempts to identify the relevance of the utilization of machine learning in the health field, especially in dementia, presenting the impact of using machine learning for disease diagnosis support. In addition, the features identification and most used methods for dementia identification were analyzed and discussed. Limitations were identified to be future explored and studied.

The paper is organized as follows. Section 2 describes the methodology used in this literature review. Section 3 presents the main results of the analysis with a subsection discussion. Section 4 provides conclusions and directions for future research.

## 2 Methodology

The chosen research technique of study is a systematic literature review [9]. This systematic literature review attempts to identify all relevant research papers to answer the research question formulated previously [10]. A combination of bibliometric and content analyses and social network methods was used for the analysis process. This state-of-the-art research describes the state of development and the demand rate for this topic over the last 20 years. The Scopus database was used for the period from 2001 to 2021.

The main research questions that this work aims to address are as follows:

RQ1: What is the relevance and the impact of ML in the health area, particularly in dementia?
RQ2: What is the aim of ML methods usage?
RQ3: What ML methods used the most in the area of dementia?
RQ4: What features are used to classify or predict dementia?

The research method can be shown through three parts, which describe the detailed process of the study, and each part is divided into a few substages.

### 2.1 Search strategy

The first part consists of substages such as *Identification* and *Inclusion*. The identification part includes preprocessing the search for corresponding publications using established keywords in a limited period. This helps determine the growing interest in dementia in machine learning and narrow the circle of results of interest. After analyzing the results of the first substage, it is possible to identify the top five keywords to continue the process in the substage “Inclusion”. Investigation of the obtained results allows the extraction of irrelevant papers by applying limitations and acquiring documents that focus only on the area of interest [9].

### 2.2 Bibliometric analyses

The next stage is the bibliometric analysis. This stage was performed using a Social Network Analysis (SNA). SNA focuses on creating maps and measures of the relationship structure to determine obstacles to information flows [11].

Bibliometric analyses include analyses of *Publication by year*, *Country analysis*, *Top keywords analysis* and *Top source journal analysis*.

The bibliometric visualization was performed by the software VOSViewer (version 1.6.18). VOSViewer software is a tool for creating visualization based on clustering techniques [12].

Bibliometric mapping is created to receive a statistical analysis of outputs (publication, citation, etc.) in the academic world.

VOSViewer shows the connection of units by generated networks and graphs, which were previously calculated automatically. A network in a map is a set of objects of interest connected between them [13]. Excel was used to collect and input data.

### 2.3 Discussion

Answers to the predetermined research questions are presented in the final subsection, along with the discussion and results. Scientific details associated with the references found are compared, analyzed, and discussed.

## 3 Results

**Figure 2:**
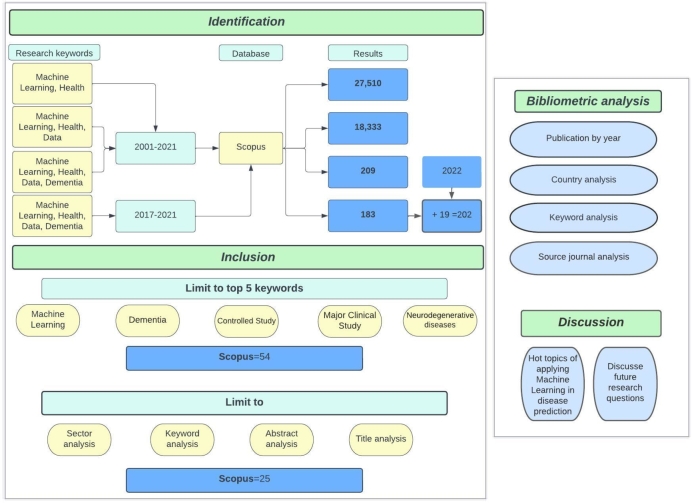
Process flow chart.

This section presents the results and analysis. The overall situation is described on Fig. 2.

The Fig. 2 illustrates preprocessing of data in subsections *Search strategy*, which divided into *Identification* and *Inclusion*, as well as titles of different analysis in subsection *Bibliometric analysis* and inclusion of subsection *Discussion*.

### 3.1 Search strategy

The first stage involved searching for and collecting related materials. The following subsections present strategies adopted for identifying and including scientific papers.

The criteria for selecting papers included:

- Papers selection including selected keywords defined on the identification stage.
- Analysis of the headlines of papers for the relevance of the study topic.
- Analysis of papers abstracts checking their conformity to the research topic.

#### 3.1.1 Identification

This phase considered searching for materials to collect documents related to artificial intelligence and medicine. The first step is the identification performed using the Scopus database to conduct an extensive review. In addition, Boolean operators have been used to save time from inappropriate matches [9]. Initially, it was decided to use primary keywords that describe the general idea of research, “Machine learning” OR “Health.”

The search was conducted to understand the topic's popularity over a time interval from 2001 to 2021. Since the area of interest was established in disease prediction of dementia by applying artificial intelligence, particularly machine learning techniques, two keywords were chosen for the first step.

The results of this submission show 27,520 papers (in May 2022). For the last 2021 year, 8 852 studies were published, but in 2016, the number of studies reached 1019, which shows an increase of 869%. This describes the recent growing interest in using ML techniques for disease prediction (Table 1).

**Table 1:** Results of the Scopus database for 2021 year.

Field of search	Number of papers
Machine Learning, Health	8852
Machine Learning, Health, Data	5748
Machine Learning, Health, Data, Dementia	63

**Figure 3:**
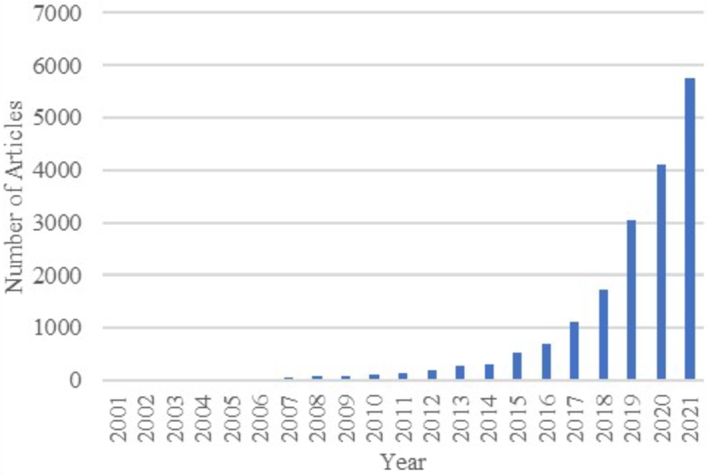
Results of search on the Scopus database by using keywords: Machine Learning, Health and Data. The chart shows the progress of the number of publications for each year.

The next attempt specifies more searches by adding keyword such as “Data” to the previous “Machine learning” OR “Health.” This method was used to conduct a thorough analysis of the relevance and development of the application of machine learning methods in the health field and, more specifically, to identify dementia. The total obtained result was 18,333 documents, and the first boost was noticed in 2017, starting with 1124 papers and continuing with 5748 studies in 2021. Again, proving the fact (Fig. 3) that after a long period of slight growth, which was maintained until 2016, the attraction of the theme was reactivated.

Working with this large number of documents is a challenge; therefore, to narrow the circle of publications for systematic literature analysis, the keyword “Dementia” was added to the previous set of keywords “Machine learning,” “Health,” and “Data.” The keyword “Dementia” identified a necessary sector of the requested search and returned 209 files for the past 20 years.

As mentioned before, the first noticeable rise in interest in the topic of machine learning combined with the health field was noticed in 2017, and the research has grown since then. So, the same set of keywords was used to identify the number of publications for the last five years from 2017 to 2021; the final result was 183 works. However, because this review was written in the first part of 2022, it was fair to include documents of completed years and files from 2022 (183+19=202 publications for five months of 2022 year), considering only the future work description process to be informed of the latest innovations [9].

#### 3.1.2 Inclusion

After completing identification, the next step was the inclusion/exclusion process. This stage involves the selection process of documents, in which outcomes in the final set of publications are addressed to perform bibliometric analyses [9]. First, we analyzed the returned 202 files for the top five most repetitive keywords. They turned out to be the leading search keywords, such as *“Machine Learning”, “Dementia”*, and new *“Controlled Study”, “Major Clinical Study”, “Alzheimer diseases”, and “Neurodegenerative diseases”*, shown in Table 2, with the purpose of going to the next step of inclusion/exclusion.

**Table 2:** Most repeated keywords for conducting the inclusion stage.

Keywords	N0 repetition
Machine Learning	140
Dementia	117
Controlled Study	54
Major Clinical Study	48
Alzheimer diseases	46
Neurodegenerative diseases	45

**Figure 4:**
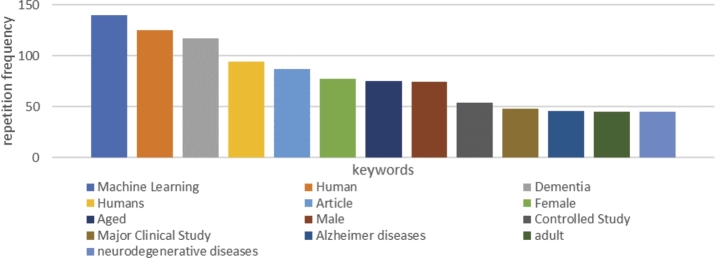
The frequency of commonly used keywords according to the Scopus database.

According to the results of the keyword examination (shown in Fig. 4), a new search on the Scopus database was initiated. Filters such as the five mentioned keywords and the period from 2017 to 2022 were applied to the investigation. This attempt reimbursed 54 documents, thus reducing the previous sum of the files. Papers that fulfilled specific exclusion criteria were excluded.

Therefore, in the last sample of inclusion, the Scopus had 25 papers that satisfied our requirements. The criteria for exclusion were:

- The paper's topic title inconsistency.
- The dissimilarity of the complete text, taking into account the abstract, keywords, and results.

### 3.2 Bibliometric analyses

This section shows the review's findings and is presented in the separate subsections.

#### 3.2.1 Publication by year

Fifty-four studies were included in this analysis, and a total of 54 studies were considered. All the original articles that participated in the selection techniques were published between 2001 and 2022. However, as mentioned above, in section 3.1.1, papers from 2022 were included to track new proposals from authors.

The annex (Table 5) presents the studies published until 2016. It can also be noticed that searching for papers with the keywords “Machine learning” OR “Health” turned the result in a large number of studies than searching for papers with the keywords “Machine learning”, “Health”, “Data”, and “Dementia”.

**Figure 5:**
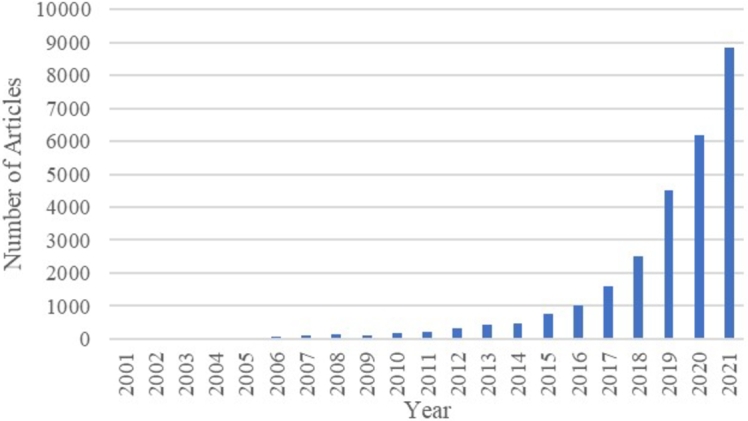
Results of search on the Scopus database by using keywords: Machine Learning and Health. The chart shows the progress of the number of publications for each year related to machine learning in the health area.

Fig. 5 shows the irrelevance of AI applications for health problem solutions in the early 2000s.

**Figure 6:**
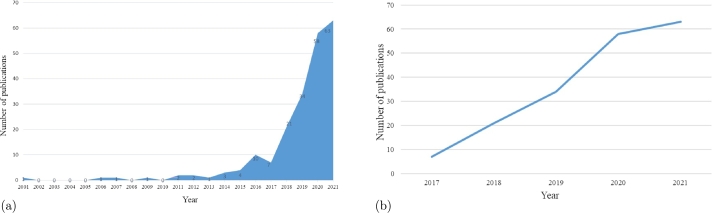
Results of annual publications on the Scopus database using the following keywords: Machine Learning, Health, Data and Dementia: (a) for the past 20 years; (b) for the past 5 years.

Fig. 6(a) demonstrates the deficiency of studies on AI in dementia prediction in the first decade of the relevant period. However, the present time could be discovered as a rebirth of AI owing to high interest and a huge range of publications each year [12]. On the Scopus database, in total 26 documents had been registered for the first 15 years, wherein become mentioned word “dementia”, is approximately one publication per year. However, this situation changed after 2017, as shown in Fig. 6(b), where the boost was recorded with the growth rate equal to 200% in the 2017-2018 years. In the following years, the increase continued, but not at a large rate. The high percentage of growth could be explained by an increased mortality rate from Alzheimer's, especially after age 65 in the period 2000-2018 years, according to paper [14]. Annually, more than ten newly approved scientific papers appear and will likely continue with the same haste.

Ultimately, the number of published documents associated with our subject matter has increased over the past five years, demonstrating that the interest in using ML in health lies in the ascending trajectory and still has research subjects.

#### 3.2.2 Country analyses

This study used VOSViewer software to analyze 54 studies to comprehend relations between countries and authors through a scientific map and recognize countries that contribute the most to the application of ML in health. Forty-eight counties participated in the analysis. The data were extracted from the Scopus database and used to create the bibliometric network. The type of analysis is co-authorship, and the unit is the country. The threshold for the minimum number of documents for a country is one. The network graph was generated based on the set parameters, with the previously determined total strength of co-authorship links with countries.

**Figure 7:**
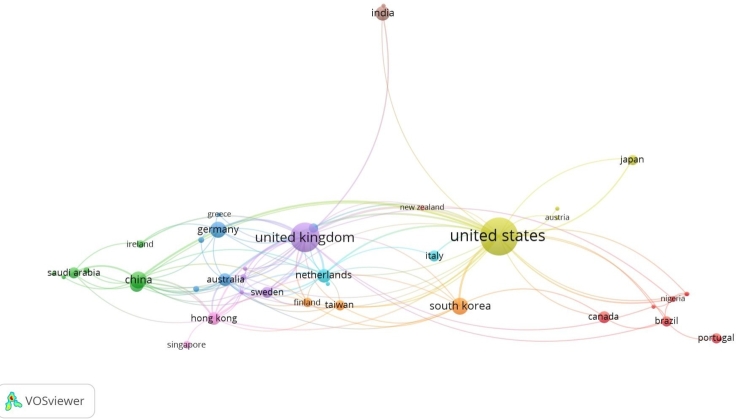
Bibliometric map based on the network of co-authorship relations among 48 countries.

The Fig. 7 is the bibliometric map describing network among 48 countries of international co-authorship.

In this visualization, the unit is the country, and they are specified as labels and circles. The more important a country, the larger its label and its circle relatively. The size of the circle is proportional to the significance of the country and the larger number of articles published by the authors from that country. The lines connecting circles represent collaborations between organizations in these countries [13]. The thickness of the lines describes the number of publications, and an extended distance can show a weak similarity of works. Each circle has its own color and a set of similar color circles close to each other built cluster. Consistent with the clustering technique, VOSViewer splits into 10 clusters of different colors (yellow, purple, green, blue, orange, etc.), and there are large and small clusters [12].

It is clear from the map, that two clusters (yellow and purple) with spread connections and grander than the others. The yellow cluster represents countries with works linked to the United States (28,82%), and the next purple cluster illustrates works with the United Kingdom (25,88%). After the last two countries, one more European country might be mentioned, such as the Netherlands (14,12%) with 24 international collaborations, whereas Germany has 12 total links strengths and Portugal has two international collaborations. The leader among Asian countries is China (13,53%) with 23 total link strengths, followed by Hong Kong (22 links), and South Korea (12 links).

#### 3.2.3 The top keywords analyses

For the analysis, 202 articles were used to search the titles and abstracts for keywords by VOSviewer software. Each same color association means the cluster.

**Figure 8:**
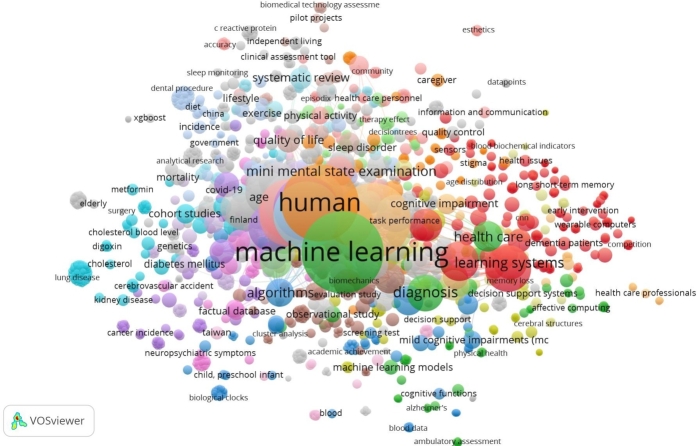
Bibliometric map based on the network of keyword's co-occurrence among 202 publications.

In the map in Fig. 8, the high-frequency keywords were machine learning (140), human (125), dementia (117), article (87), female (77), aged (75), male (74), controlled study (54), major clinical study (48), Alzheimer's disease (46), adult (45), and neurodegenerative diseases (45). Keywords determining hot topics include machine learning, dementia, controlled study, major clinical study, Alzheimer's disease, and neurodegenerative diseases. This set of keywords participated in the selection process and helped establish the final research set.

#### 3.2.4 Top source journal analyses

**Figure 9:**
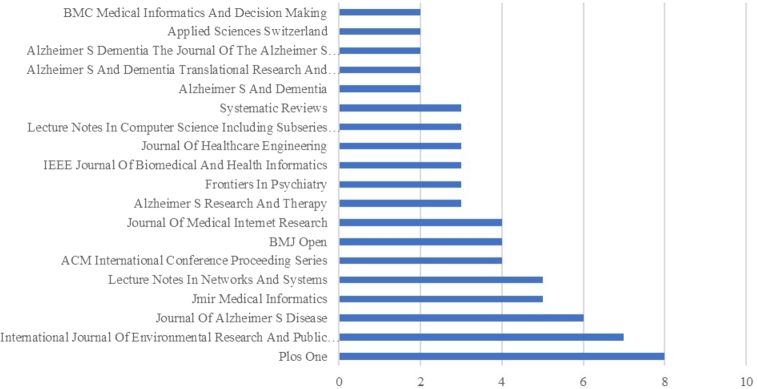
Top 20 source journals.

This section considered the top 20 sources from the final papers with the highest published frequency. Overall, 202 studies were published in 137 publication sources or journals. Among the 137 sources, only 32 journals had more than one paper, whereas approximately 105 sources were detected in one article. The first 20 sources were considered for constructing diagrams with more than 1 to 8 published papers. The total percentage contribution of all 20 sources is approximately 36,14%. Fig. 9 shows that the top source journals are *Plos one (8 papers; 3,96%), International Journal of Environmental Research and Public Health (7; 3,47%), Journal of Alzheimer S Disease (6; 2,97%)* of total. After this top, following with five papers, journals, such as *Jmir Medical Informatics and Lecture Notes In Networks And Systems* have 2,5% contribution. Next, nine sources had four or three papers per each, with approximately 2% of the total, whereas the rest had two papers, and the publication contribution was less than 1%.

### 3.3 Discussion

In this systematic review, we identify 25 studies that used neuroimaging data intending to answer the four research questions established at the beginning of the review.

#### 3.3.1 RQ1: the relevance and the impact of ML in the health area, particularly in dementia

According to the findings listed in the *Results* section, there has been a noticeable rise in interest in using machine learning techniques in the field of health from the start of 2017 to the present, proving the topic's high relevance. This increase in interest can also be seen in the use of machine learning to diagnose dementia, which has seen a sharp rise in publications on the subject over the previous five years.

#### 3.3.2 RQ2: the aim of ML methods usage

In this study, [15], investigated the possibility of classifying AD patients among 158 patients with MCI or dementia of various causes based on a neuropsychological test (NPT). The algorithm Support Vector Classifier was used to predict AD based on the results of the tests conducted and achieved high accuracy, 82-89%. Another study [16] aimed to identify morphological changes in brain tissue caused by AD and set it up as a diagnostic marker. The authors developed techniques for automatically detecting and segmenting the corpus callosum. 1437 MRIs were applied in a multivariate pattern analysis using SVM.

A study [17] presented the ability of the framework to detect MCI patients and from MCI to AD patients with modality T1-weighted MRI and achieved accuracies of 69% and 75%, respectively. The authors of [18] tried distinguishing AD and MCI by developing an ensemble of 3D-CNN and ADNI-T1-weighted MRI. Classifying AD, MCI, and HC (Health Control) has also been attempted in study [19], but with a novel multi-model ML system using the EEG of the patients. Three different feature sets were first extracted and then tested through Multi-Layer Perceptron (MLP), SVM, Autoencoder (AE), and Logistic Regression (LR) techniques. The proposed approach showed the best results, and the MLP architecture outperformed the other models in classifying AD, MCI, and HC with results of 89,22%.

Studies [5], [20], [21] and [22] aimed to discriminate between or predict only patients with MCI. In [5], the deep learning (DL) approach was applied to classify cognitive normal (CN) and two types of MCI (Early MCI and Late MCI). Using DL to extract features from MRI helps obtain results of 93%. Study [20] proposed a computational model based on an ANN to determine the necessary parameters for the proper diagnosis of MCI. The analysis showed that using cognitive tests produced an accuracy greater than 90%. The EGG recording system is considered attractive due to the low cost, mobility, and possibility of multiple scanning. The type of study [20] and [23] explores data mining and clustering. The study [22] created a new approach to distinguish patients with MCI using EEG signals. The method is based on a dictionary learning algorithm, CLC-KSVD classifier. Dictionary learning is a tool for analyzing medical signals minutely. Algorithm K-SVD (K-means and Singular Value Decomposition) is a clustering-based method where the cluster center is a dictionary atom; CLC-KSVD (Correlation-based label consistent KSVD) is a recent classification approach of dictionary learning. 61 EEG signals were investigated, and the proposed method CLC-KSVD presented the best results, with 88,9% accuracy, in a few brain regions: channels in the temporal and left zones and the left temporal zone. In survey [21], authors developed three models based on three ML techniques (ELM, SVM, KNN) that can distinguish MCI from EEG data. As a result, Extreme Learning Machines (ELM) presented the best performance of 98,78% in a short time.

Several studies have focused on investigating metabolic patterns [24], [25] and biomarkers [26], [27], [28], [29]. The study [24], used a pre-trained machine learning-based AD designation (MAD) framework to compare AD-related metabolic patterns in 27 patients with dementia and 20 non-dementia people, all 47 participants had Positron Emission Tomography (PET) scans. The MAD algorithm displayed 18/27 dementia patients have dementia, which created future research as to whether the different types of dementia have AD metabolic patterns or not, which would be interesting for study. The research [25], tested the efficiency of plasma metabolites in identifying AD in cognitively normal individuals (CN). DL and Ensemble methods were used for 242 CN people and 115 AD individuals. The XGBoost method presented better results than the others and led to new future work, indicating the specificity of metabolites for other neurodegenerative disorders.

In [27], the authors utilized PET and T1-weighted MRI of 79 persons to classify with SVM algorithm dual PET biomarkers and also compare results of brain sections. The results revealed interesting outcomes for future studies; SVM displayed certain feasibility in detecting AD in the temporal cortex. Seven ML algorithms were applied (Decision Tree, Elastic Net, SVM, RF, K-NN, Gaussian, and Multinomial Naive Bayes) in study [28], for testing EEG as a biomarker for diagnosing primary progressive aphasia. The same goal followed work [26], but discover significant biomarkers single nucleotide polymorphisms for AD disease. As algorithms combine SVM-based classifiers with kernels, Naive Bayes, and K2 learning algorithms, among others. The authors explore two databases, named, ADNI with 757 subjects and WGS (Whole Genome Sequencing) - with 812 subjects. The better effectiveness was obtained by Naive Bayes and K2 methods. In study [29], the authors found a combination of biomarkers that could serve as a key biomarker panel for early AD detection. The SVM algorithm achieved a sensitivity of 80%.

In study [30], the fMRI voxel profiles are proposed to solve the automatic analysis pattern of people with dementia and non-dementia. The joint combination of four different voxel selection techniques (Random feature elimination (RFE), ReliefF, Activation sum, Activation per class) with six pattern recognition algorithms (SVM, SMO, LibLinear (large-scale linear support vector regression), Naive Bayes, Random Forest, Random Subspace, Linear Oracle) used. This experiment yielded promising results with a 95% accuracy, highlighting SMO and RFE. Another study [31] identified language impairments in patients with AD. Instead of modality, researchers used a picture description task of 154 participants from two languages (106 English speaking and 47 French speaking) and applied natural language processing (NLP) techniques with ML. Research has shown that language impairments in AD can be observed in everyday speech.

The different types of studies, their goals, and the selected features are listed in Table 4. It shows that ten out of eighteen work types is the classification of Alzheimer's disease, which is much greater than studies predicting AD.

Machine learning can enhance the speed and efficacy of the diagnostic process. As mentioned earlier, timely diagnosis detection can significantly benefit patients and their families in managing the disease trajectory. Machine learning facilitates the efficient handling of vast quantities of clinical data, for instance, neuroimages, biomarkers, etc, thereby ensuring high precision and rapid analysis. In contrast, human analysis of large datasets can be arduous and time-consuming, leading to the risk of overlooking critical details. Furthermore, acquiring high-quality big data poses a formidable challenge, limiting research endeavors.

#### 3.3.3 RQ3: the most used ML methods in the area of dementia

The literature survey highlights three widely used machine-learning algorithms among the reviewed papers: Support Vector Machine (SVM), Ensemble Methods, and Convolutional Neural Network (CNN), as shown in Table 3.

**Table 3:** An overview of the related studies.

Algorithm	Reference
Support Vector Machine	[32], [33], [28], [31], [21], [34], [35], [27], [20], [26], [30], [36], [29], [16], [37], [19]
Ensemble Methods	[32], [35], [28], [20], [25], [30]
Convolutional Neural Network	[5], [18], [32], [33], [23], [37]
K-Nearest Neighbor	[32], [28], [21]
Naive Bayer	[28], [30], [26]
Decision Tree	[32], [28]
Linear programming	[31]
Gaussian process classification	[35]
Import Vector Machine	[34]
Regularized Extreme Learning Machine	[34]
Artificial Neural Network	[20]
Dictionary learning	[22]
Others	[15], [31], [17], [24], [19], [38]

SVM is the most used classifier in studies is SVM researchers' preference can be explained by the study's aim to identify whether a patient is healthy or not, pursuing binary classification.

The following most popular methods are Ensemble Methods, including Random Forest, Extreme Gradient Boosting (XGBoost), Adaptive Boosting (AdaBoost), and Stochastic Gradient Boosting. Random Forest was in demand more than others because of its good performance and combination ability and was applied in four research works.

There are several applied architectures of CNN algorithms, such as Class Activation Mapping (Grad-CAM), Convolutional Autoencoder, Ensemble of 3D convolutional networks, 3D-DenseNet, 3D Very Deep Convolutional Network (VGGNet), and 3D Deep Residual Network (ResNet). This leads to the conclusion that the use of deep learning is accelerating.

#### 3.3.4 RQ4: features are used to classify or predict dementia

Most studies based on clinical features, such as MRI and PET, provided more exact information and contributed to accurate results. Almost all studies included MRI data and their different types, such as structural MRI (sMRI), functional MRI (fMRI), T1 - weighted MRI. As additional data, demographic and clinical data were applied, such as age, sex, educational level, and MMSE score, and several studies presumed information about the number of patients with and without diseases.

Most studies chose The Alzheimer's Disease Neuroimaging Initiative (ADNI) as the database. The ADNI website includes several data types, namely clinical and genetic data, MRI and PET images, and biospecimens. According to this work, research is focused on using MRI and PET images; however, different data that can be explored, Diffusion Tensor Imaging (DTI), multi-echo Gradient Recalled Echo T2*-weighted imaging (GRE T2*WI), Fluid-Attenuated Inversion Recovery (FLAIR), or Arterial Spin Labeling (ASL).

From the considered studies, the authors' limitations and difficulties were the data's availability and preprocessing. The complication of acquiring original high-quality data and possessing more extensive independent data could improve the efficiency of the results. The data were divided into test and training sets to evaluate the ML model. The training set was used to train the model, and the test set was used to show the performance of the final model. Lack of access to a large amount of data can interrupt this process, thus formulating a plan for future research. Another preprocessing problem is the technical variability in MRI scans due to images taken from different scanners, head motions, and different positions, all of which can affect the algorithm's capacity.

## 4 Conclusions and future work

This systematic literature review provides a concept for the state-of-the-art applications of AI and ML in health, particularly in dementia prediction. A total reviewed 25 studies was reached by identifying 27510 papers related to ML in health over 20 years (2001-2021). The analysis concerned the distribution of annual publications, study countries, most keywords, and source analyses. Due to its extensive database in machine learning integrated with health-related research, the Scopus is preferred as a more complete resource for scholarly endeavors.

The findings from this literature review reveal that research on ML in dementia is a promising subject. However, the literature on the application of ML algorithms to neurodegenerative diseases is limited compared to the literature on AI in health. Considering that the clinical problem of early prediction/diagnosis of neurodegenerative diseases is always relevant, this topic remains unexplored.

In addition, this review provides suggestions for future research. The authors investigated specific themes to predict the progression of dementia. According to the literature survey, in the case of the objective type, AD classification prevails in comparison with the prediction of MCI or a combination of MCI and AD.

Study limitations were defined as small data sizes, quality, and preprocessing steps. Future work will consist of continuing research based on the results obtained, investigating it in greater detail with better and varied data types, such as cognitive and social data, exploring various techniques and expanding the diagnostic range, considering other forms of dementia rather than just AD, and attempting to apply the findings.

The SVM is the most commonly used classifier for identifying AD and MCI. The following two methods are Ensemble Methods and CNN. Also, studies have used a combination of several algorithms. The least researched theme with more open issues for work is evaluating and discovering new biomarkers and metabolic patterns and testing new features for predicting neurodegenerative disorders.

Table 4 shows that MRI and its types were the main features for almost all research, accompanied by MMSE scores and demographic features, such as age, gender, and educational level.

**Table 4:** Resume of selected studies.

Ref.	Type	Aim	Features
[5]	Classification MCI	Identify healthy people and the two types of MCI	sMRI, PET, and biological markers
[18]	Classification AD, MCI	Identify AD and MCI using brain MRI	T1-weighted MRI
[15]	Classification AD	Identify AD patients from MCI or dementia patients	CSF, tau/A(1-42) ratio, TB ratio, MMSE
[32]	Classification AD	Diagnose of AD	fMRI, MMSE
[34]	Classification AD	Diagnosis of AD progression	sMRI
[33]	Prediction AD	Building three ML classifiers to predict AD	T1-weighted MRI
[35]	Classification AD	Classify early diagnosis of AD	T1-weighted MRI
[28]	Prediction PPA	Evaluating EEG as a biomarker of diagnosis	MRI, FDG-PET
[27]	Classification AD	Classify AD diagnosis in small sample sizes	PET (18F-FDG and 11C-PiB) and T1- weighted MRI
[17]	Prediction MCI, AD	Predicting MCI and AD prior to the clinical events	MRI
[20]	Data Mining	Analysis of data parameters to achieve high accuracy in neurodegenerative disorders diagnosis	gender, age, education, study time, MMSE, SVFT, CDR and AD8
[31]	Classification AD	Identify AD related language impairment	Picture description task (syntactic, semantic and paralinguistic features)
[21]	Classification MCI	Identify MCI patients from healthy control subjects	EEG
[23]	Clustering	Find patterns between cognitive symptoms and the underlying neurodegeneration process	T1-weighted MRI, MMSE
[25]	Classification AD	Characterise AD base on plasma metabolities	metabolite data
[24]	Classification AD	Identify AD-related metabolic pattern	FDG-PET, MMSE, CT, MoCA, FAB
[30]	Prediction AD	Using of fMRI voxel profiles to tackle the automatic pattern analysis of AD	fMRI
[36]	Classification AD	Predict conversion from MCI to AD by integrating new features	sMRI, rs-fMRI, MMSE, age, CDR and FAQ scores
[26]	Classification AD	Predicting AD by discovering new biomarker	SNPs biomarker
[16]	Classification AD	Identify morphological changes of brain tissue caused by AD and establish it as a diagnostic marker	T1-weighted MRI, CDR, MMSE, GDS
[29]	Classification AD	Identify potential biomarkers for early AD detection	biomarkers
[38]	Classification AD, MCI	Developing an automatic technique for classifications of MCI and AD	T1-weighted MRI, age, gender, MMSE
[22]	Classification MCI	Identify MCI patients by using EEG signals	age, education, EEG, NUCOG, MMSE
[19]	Classification AD, MCI	Classifying EEG recordings of AD and MCI individuals	EEG
[37]	Classification AD	Multi-class classification of AD and MCI subjects	sMRI

Notation: MCI - Mild Cognitive Impairment; AD - Alzheimer's disease; MRI - Magnetic Resonance Imaging; PET - Positron Emission Tomography; EEG - Electroencephalography; MMSE - Mini-Mental State Examination; CSF - Cerebrospinal fluid; SVFT - Semantic Verbal Fluency Test; CDR - Clinical Dementia Rating; AD8 - Ascertaining Dementia; MoCA - Montreal Cognitive Assessment; FAB - Fragment antigen-binding region; CT - Computerized Tomography; FAQ - Functional Activities Questionnaire scores; SNPs - Single nucleotide polymorphisms; GDS - Global Deterioration Scale; NUCOG - neuropsychiatry unit cognitive assessment tool.

## CRediT authorship contribution statement

**Gauhar Kantayeva:** Investigation, Writing – original draft. **José Lima:** Supervision, Writing – review & editing. **Ana I. Pereira:** Supervision, Writing – review & editing.

## Declaration of Competing Interest

The authors declare that they have no known competing financial interests or personal relationships that could have appeared to influence the work reported in this paper.

## Figures and Tables

**Table 5:** Distribution of publications over 20 years.

Keywords	Machine Learning, Health	Machine Learning, Health, Data	Machine Learning, Health, Data, Dementia
2001	15	13	1
2002	12	6	
2003	23	10	
2004	30	20	
2005	35	17	
2006	61	35	1
2007	92	53	1
2008	137	87	
2009	117	75	1
2010	172	101	
2011	222	139	2
2012	318	205	2
2013	426	268	1
2014	455	303	3
2015	742	536	4
2016	1019	702	10
2017	1603	1124	7
2018	2491	1736	21
2019	4498	3056	34
2020	6190	4099	58
2021	8852	5748	63

## Data Availability

Data included in article/supp. material/referenced in article.
